# The Persian Version of Fertility Adjustment Scale: Psychometric Properties

**DOI:** 10.22074/ijfs.2018.5219

**Published:** 2018-03-18

**Authors:** Asma Tiyuri, Seyyed Abolfazl Vagharseyyedin, Marziyeh Torshizi, Najmeh Bahramian, Morteza Hajihosseini

**Affiliations:** 1 Faculty of Nursing and Midwifery, Birjand University of Medical Sciences, Birjand, Iran; 2 East Nursing and Midwifery Research Center, Birjand and Midwifery College, Birjand University of Medical Sciences, Birjand, Iran; 3 Cardiovascular Diseases Research Center, Birjand University of Medical Sciences, Birjand, Iran

**Keywords:** Adjustment, Fertility, Infertility, Iran, Psychometrics

## Abstract

**Background:**

Infertility is a common clinical problem. Psychological adjustment to infertility refers to changing the viewpoint and attitude of an infertile person toward infertility problems, treatments and possible outcomes. The present study aims to prepare a valid and reliable scale for assessing the psychological adjustment to infertility, by determining the cultural adaptation, validity and reliability of the Persian version of the Fertility Adjustment Scale.

**Materials and Methods:**

This is a cross-sectional study performed to localize and validate the Fertility Adjustment Scale, in which 40 infertile women and 40 healthy subjects (fertile or having children) were detected by a gynecolo- gist and the subjects who completed the Fertility Adjustment Scale (FAS) questionnaire were recruited. This study had four steps: in the first step, the literature was reviewed, in the second step, the scale was translated, in the third step, the content and construct validity indicators were calculated, and in the fourth step, reliability of the scale was validated.

**Results:**

The mean (± SE and range) of fertility adjustment total scores in the infertile group and the control group were 43.2 (1.2 and 27-57) and 42.3 (1.5 and 18-57), respectively (P=0.623). The content validity was good according to Con- tent Validity Index score (0.7-0.8). A two-component structure was extracted from factor analysis which approximately justifies 52.0% of the cumulative variations. A Cronbach’s alpha value of 0.68 showed moderate reliability.

**Conclusion:**

The results of this study revealed that the infertility adjustment scale is a useful tool for the analysis of psy- chological reactions towards infertility problems and evaluation of the consequences of treating this social-clinical problem.

## Introduction

As a natural stage of life and one of the most important aims of each marital bond, fertility, reproduction and breeding are considered the basis of human survival (1, 2). Fertility is a physiological process in living creatures, which also involves social and mental dimensions of human life (2). Infertility is defined as the inability to get pregnant after one year or more of regular unprotected sexual intercourse during the menstrual fertility cycles, without using contraceptive methods (1, 3, 4).

Infertility is a common clinical problem which, as estimated by the World Health Organization (WHO), has affected about 60 to 80 million couples all over the world and its prevalence is estimated to be around 10 to 15%, worldwide. Meanwhile, this problem is more prevalent in developing countries (1, 5-7). A meta-analysis on the prevalence of infertility in Iran showed that its prevalence was about 13.2% in Iran, the lowest rate being 2.8% in 2001, and the highest being 24.9% in 2010 (8). Also, a study in 2015 found a prevalence of 17.3% for primary infertility in Iranian couples (9).

Having the features of a traumatic event including the length of time, complicated condition, unpredictability and uncontrollability, infertility creates a full-scale crisis in the lives of infertile couples, and has been described as a global health problem with physical, mental and social dimensions (6, 10).

The person who is not able to have children or experi. ence the natural reproduction process is called “infertile” and this may trigger psychological problems especially in the Iranian culture where parents and relatives have a key role in the couple’s life; under this condition, infertility can be considered as one of the worst experiences of life (6, 7, 10). Infertile people experience depression, grief, fear, inefficiency, lack of control, and high levels of anxi. ety and guilt, and they are concerned about their body and sexual function disorders all of which are the symptoms of lack of fertility problems adjustment (6, 11, 12).

Most Iranian infertile couples face a type of stigma and consider themselves as a misfit in the community due to the inability to have their own children (13, 14). Sultan and Tahir (7) studied the psychological consequences of infertility in 400 couples (200 fertile and 200 infertile couples), samples were randomly selected from different cities in Pakistan. The results showed that fertile couples have lower levels of depression, aggression and anxiety, but higher levels of self-esteem and marital satisfaction than infertile couples. Since most infertile couples, especially those under-treatment, are reported to have psychological problems, the couples’ psychological problems should also be considered in medical treatments and interventions (13, 15).

Adjustment to infertility refers to changing the viewpoint and attitude of an infertile person towards infertility problems, treatments and possible outcomes. Fertility Adjustment Scale (FAS) was introduced by Glover et al. (14) to evaluate the psychological adjustment to fertility. The results showed satisfactory reliability and validity of the scale. In addition, Arslan and Okumus (16) localized a Turkish version of the fertility adjustment scale. The study was carried out on 240 women with infertility who referred to the infertility center of a hospital in Turkey.

In this scale, adjustment is defined as an appropriate management of behavioral, mental and emotional responses to infertility (11, 14). Adjustment does not mean that the couple do not have the desire to have children anymore or to accept their current situation, rather it reflects the extent to which the couple are able to cognitively, emotionally and behaviorally process the possibilities of having or not having children and how to get prepared for both situations (14).

After evaluation of the validity and reliability of this scale, it has been used for investigating the psychological consequences of infertility treatment in infertile couples (11, 13, 15). To the best of our knowledge, despite the importance of adjustment to fertility, no study has been conducted in this regard in Iran. Hence, the present study aims to prepare a valid and reliable scale for assessing the psychological adjustment to infertility, by determining the psychometric properties of the Persian version of FAS.

## Materials and Methods

This is a cross-sectional study conducted on 40 infertile and 40 fertile women (with or without children) in Birjand, east of Iran from November 2016 to January 2017. Fertility/infertility was diagnosed by a gynecologist, and the participants were selected from the available subjects who completed the FAS. 

Given that there has been no similar study in Iran, and the desirable conditions for conducting the pilot study were not known, hence the sample size was estimated with a sensitivity of 0.85 and a specificity of 0.70. Also, as there was no infertility center in the city of Birjand, the patients and healthy subjects (women with a history of having children) were selected from those referring to obstetricians’ clinics. The inclusion criteria involved being infertile, completion of the informed consent for participation, being within the age range of 18-45 years and having the ability to read and write. According to previous studies (17, 18), there were four steps. In the first step, the literature was reviewed, in the second, the tool was translated, in the third step, content and construct validity indicators were calculated and in the fourth step, the reliability of the tool was evaluated.

### Review of the literature

In the present research the terms, adjustment, fertility and infertility were selected as the search keywords. Documents were obtained from scientific databases such as PubMed, Science Direct, Medline, Embase, Scopus and Google Scholar as well as Persian electronic resources namely, SID, Irandoc, Iran-Medex and Magiran. One of the main aims of this step was to examine the possibility of existence of a Persian version of this scale, but no such version was found.

### Translation of Fertility Adjustment Scale 

Translation and back-translation method was used in this step (19). For this purpose, the questionnaire was first translated into Persian (Farsi) independently by an English-language expert and a nursing and midwifery expert fluent in English. Afterwards, the translated versions were reviewed by two nursing and midwifery experts, and then the Persian version was translated into English by an English-language expert and a nursing and midwifery expert fluent in English. Finally, all experts reviewed and approved the compliance of the Persian version with the original one.

### Calculation of content and construct validity

In order to evaluate the face and content validity of the tool, the translated draft was handed to 2 obstetricians, 3 nursing experts and 4 gynecologists working in the Faculty of Medicine and Faculty of Nursing and Midwifery. In order to determine the content validity of the questions of the above mentioned questionnaire, the experts group was asked to judge the suitability of every question based on a 3-point Likert scale. Then, using the face and content validity indicators, the beneficial questions were selected. At this point, the content validity ratio (CVR) was calculated for each statement by the following equation (20).

$$\mathrm{CVR}=\frac{n_{e}-\frac{N}{2}}{\frac{N}{2}}$$

Where, ne is the number of the experts who considered the question as necessary and N is the total number of the experts. The obtained value for each question was then compared with the Lawshe Table criterion for 9 experts which equaled to 0.77 (21). Some of the statements and phrases were modified, and in order to have a better evaluation of the final version regarding the difficulty level, 10 questionnaires were given to infertile women, and no particular problem was observed. The Exploratory Factor Analysis (EFA) method was used in order to assess the construct validity. Given that the questionnaire was translated for the first time in Iran and the original designer did not use exploratory factor analysis to present basic model, we used EFA and believed that next studies on this questionnaire in Iran need to perform CFA based on our findings.

EFA was utilized for evaluating the presence of possible subscales and the construct validity, using the principal component analysis and varimax orthogonal rotation. In this procedure, before performing the exploratory factor analysis, the Kaiser-Meyer-Olkin (KMO) index was evaluated and Bartlett test was done. The EFA can be performed if the KMO index is >0.5 and the Bartlett test P value is <0.05. In addition, the explained variance indicators (>0.6), eigenvalues (>1) and rotated factor loadings (>0.4) were used for selecting the components (22).

### Reliability of the tool

Reliability of the questionnaire in terms of internal consistency was assessed using Cronbach’s alpha coefficient. This index ranges from 0 to 1, and values close to 1 indicate better reliability. Cronbach’s alpha of more than >0.7 reflects a good internal reliability (23).

### Fertility adjustment questionnaire details

Fertility adjustment questionnaire was developed in 1999 by Glover et al. (14). It contains 12 questions to which the participants respond and is evaluated using a 6-point Likert scale (strongly agree=6 and strongly disagree= 1). Statements with positive aspects (2, 4, 6, 9, 10, 12) were inversely coded. The minimum score is 12 and the maximum score is 72, and the fertility adjustment total score is the sum of scores. A high score means low adjustment level. The internal consistency of the scale has been confirmed with a Cronbach’s alpha coefficient value of 0.85. The split half reliability of the scale was approved with a correlation coefficient of 0.68 and Guttman coefficient of 0.8. Reliability of the scale was also approved by test-retest method with a correlation coefficient of 0.88. At the same time, validity of the scale was confirmed by evaluation of the correlation between the scale scores and the scores of the hospital anxiety (r=0.43) and depression (r=0.49) scale.

### Demographic data

The demographic information checklist included the following variables: age of patients, job of patients, length of marriage, length of infertility, length of treatment, age of mates and job of mates. These variables were compared between the two fertile and infertile groups, using Chi-square and Mann-Whitney tests.

### Ethical considerations

The Ethics Committee of Birjand University of Medical Sciences approved the present study (approval No. IR.BUMS.REC.1395.210). Afterwards, written informed consent was obtained from each patient, showing that the participants were recruited voluntarily and with full knowledge and could quit the study at any time and this would not have an impact on their treatment process. Moreover, they were informed that the information will be reported in a general manner, without revealing the patients’ personal information. Also, the written authorization was obtained from Glover et al. (14) who invented the questionnaires for the first time.

### Statistical analysis

The collected data were entered into the SPSS software, version 18 (SPSS, Inc., Chicago, IL, USA). The mean, standard deviation, percentage and indicators of reliability and validity were assessed. Moreover, normality of the demographic variables was examined, using Kolmogorov- Smirnov test, and non-parametric tests were used for comparing the two groups.

## Results

### Demographic information

A total of 80 patients divided into two groups of 40 fertile or infertile subjects who completed the FAS questionnaire. The mean (± SD) of demographic and clinical variables is reported in Table 1.

**Table 1:** Demographic and clinical variables


P value		Fertile n=40 Mean (SD)	Infertile n=40 Mean (SD)	Variable

Age of patients (Y)		28.6 (1.0)	27.6 (1.0)	0.487
Age of mates (Y)		32.1 (0.8)	31.4 (0.9)	0.592
Length of marriage (Y)		5.5 (0.6)	5.3 (0.8)	0.903
Length of infertility (Y)		3.2 (0.4)	--	--
Lenght of treatment (months)		18.7 (3.3)	--	--
Job of patients		n (%)	n (%)	
	Housewife	34 (85.0)	25 (62.5)	0.020
	Employee	5 (12.5)	6 (15.0)	
University student		1 (2.5)	9 (22.5)	
Job of mates				
	Employee	9 (22.5)	17 (42.5)	0.116
	Self-employed	24 (60.0)	21 (52.5)	
	Worker	7 (17.5)	2 (5.0)	


**Table 2:** Explanatory factor analysis


Item number and descriptor	Factor loading	Eigenvalue	Percentage of variance	Cumulative (%)

Factor 1				
5. I have made plans for a possible future life without a child^*^	0.72	2.80	23.55	23.55
6. I will always feel unfulfilled if I am unable to have my own child	0.64			
7. I think I could adjust to a future life without a child^*^	0.66			
8. I am sure that I can continue my normal life activities^*^	0.76			
10. I think life could be rewarding either with or without children^*^	0.69			
Factor 2				
1. I will continue with investigations/treatment until I succeed in having a child	0.73	1.3	16.62	40.17
3. I cannot plan for the future until I know for certain whether or not I can have a child	0.40			
4. I want a child of my own more than anything else in life	0.72			
Factor 3				
2. There are both advantages and disadvantages to having a child^*^	0.82	1.1	11.80	52.06
9. I cannot imagine a future without a child	0.51			


### Content and construct validity

Content validity was examined using CVR and the obtained values for the questions were in the range of 0.7 to 0.8. Construct validity was assessed using EFA. The results showed that the KMO index was about 0.68, Bartlett’s Chi-Square test result was 126.0, and the P value was less than 0.001. The results indicated the sufficiency of samples to perform this procedure. A total of 3 eigenvalues was more than 1, which justifies approximately 52.0% of the cumulative variations. Since the orthogonal varimax rotation method was utilized, factors with non- shared components were identified. All factor loadings were greater than 0.3. The first factor involved questions 5, 6, 7, 8 and 10; the second factor involved questions 1, 3 and 4; and the third factor involved questions 2 and 9, as explained in Table 2. The correlation coefficient between the first factor and the second and third factors were 0.12 and -0.02, respectively, and the correlation coefficient between the second and the third factors was -0.01. Spearman’s correlation coefficient showed no significant relationship (Table 2).

### Reliability

Reliability of the tool was evaluated using two methods. First, the correlation between the statements and the total score was evaluated and the statements with low insignificant correlations were excluded. In the second method, a Cronbach’s alpha coefficient value was utilized to show the internal consistency of the tool. According to the first method, statement 7 had low correlation (0.14) with the fertility adjustment total score which was not statistically significant. Besides, Cronbach’s alpha coefficient value for the questionnaire was 0.62, which was promoted to 0.65 after eliminating statement 7. Following the elimination of statement 4, Cronbach’s alpha value increased to 0.68, which is close to the 0.7 criteria.

### Fertility Adjustment Total Score

The mean (± SE and range) of fertility adjustment total scores in the infertile group and the control group were 43.2 (1.2 and 27-57) and 42.3 (1.5 and 18-57), respectively (P=0.623). Also, the mean (SD) of each of the statements for the infertile group is reported in Table 3. In the infertile women group, no significant correlation was observed between the fertility adjustment total score and the age, age of the mate, length of marriage, length of infertility (year), and length of treatment (month). Furthermore, no relationship was observed between the fertility adjustment total score and the job of patients and job of mates (Table 4).

**Table 3:** Mean ± SE item scores and item-to-total correlations for the Total score of Fertility Adjustment Scale


Item	Mean (SE)	Item to total correlation

1. I will continue with investigations/treatment until I succeed in having a child	5.7 (0.1)	0.40
2. There are both advantages and disadvantages to having a child^*^	2.6 (0.2)	0.26
3. I cannot plan for the future until I know for certain whether or not I can have a child	3.8 (0.3)	0.43
4. I want a child of my own more than anything else in life	5.3 (0.2)	0.44
5. I have made plans for a possible future life without a child^*^	4.8 (0.2)	0.43
6. I will always feel unfulfilled if I am unable to have my own child	4.7 (0.2)	0.76
7. I think I could adjust to a future life without a child^*^	4.9 (0.2)	0.52
8. I make sure that I carry on with my normal life activities^*^	3.8 (0.3)	0.70
9. I cannot imagine a future without a child	4.4 (0.3)	0.61
10. I think life could be rewarding either with or without children^*^	2.8 (0.3)	0.63


*; Reverse-scored

**Table 4:** Correlation of demographic and clinical variables with Total score of Fertility Adjustment Scale


Item	Correlation coefficient	P value

Age of patient	-0.11	0.325
Job of patient	-0.06	0.548
Age of husband	-0.12	0.275
Job of husband	0.008	0.941
Marriage time	-0.03	0.782
Infertility time	0.12	0.447
Treatment time	0.10	0.520


## Discussion

Infertility creates a full-scale crisis in infertile couples lives, and it has been described as a global health problem with physical, mental and social dimensions. Having a tool for evaluation of the maladjustment of spouses with respect to pregnancy is of great importance. This useful tool can be used to measure the maladjustment of infertile women or men (14). The results of the present study showed that family adjustment tool along with the remaining questions can be a trustworthy scale for measurement of infertility in in Iranian population. For attaining adjustment, a change in the patients’ behavior, emotion and recognition of their position should be made. For some patients, passing of time, studying and treatment accelerate the adjustment, while for some other patients hoping to have a baby prolongs the patients’ adjustment process.

Evaluation of the correlation of each item with the total adjustment score using Cronbach’s alpha coefficient showed that by eliminating the two questions of "I cannot talk to my husband about the possibility of not having a baby" and "I feel like I am losing my life month by month", the reliability of the tool reached the acceptable level of 0.68 and finally we had a 10-question 3-component reliable scale for measuring the adjustment to infertility. Arsalan and OKUMUS (16) indicated two components for Turkish version of FAS with 10 questions by factor analysis method with Cronbach's alpha values of 0.80 and 0.71. In addition, Glover et al. (14) as the original designer, presented a 12-question FAS with a Cronbach’s alpha value of 0.85 without performing EFA method.

Due to the time constraints and lack of sufficient human resources, it was not possible to collect data from all patients. Almost all participants in this study completed the questionnaire. The subjects were recruited from patients referring to specialists’ offices; hence, it can be said that our subjects formed a representative sample.

Considering the problems faced by infertile couples and the Iranian culture unique features on the issue, nowadays, the consequences of infertility have attracted special attentions, and tools like FAS can be useful for evaluating the psychological problems caused by infertility. Based on the obtained CVR for different items and the conducted exploratory factor analysis, this tool can be considered a valid scale for screening individuals referring to clinics for receiving psychological counseling on infertility problems. As a clinical tool, it can be the starting point for the couples’ psychotherapy sessions, which somewhat specifies the way they look at the infertility issue.

In this pilot study, the infertility adjustment tool was given to 80 patients in two groups of 40 fertile and 40 infertile women. The results showed that in the infertile women group, no significant correlation was observed between the fertility adjustment total score and the patient’s age, age of the mate, length of marriage, length of infertility (year), and length of treatment (month). Furthermore, no relationship was observed between the fertility adjustment total score and the job of patients and job of mates. Lack of relationship between this score and the mentioned demographic variables indicated that this scale is indeed a useful tool for measuring the psychological adjustment. Results were similar to those reported by Arsalan and OKUMUS (16).

Further clinical studies on larger population are needed to standardize the information obtained by using this tool in different medical centers. It is suggested that this questionnaire should be used in infertility treatment centers as the first step to provide the couple’s with a better understanding of each other’s point of view, and also an initial screening for the physician and health care team to understand psychological status of patients.

## Conclusion

The results of this study revealed that Persian infertility adjustment scale is an appropriate tool for the analysis of psychological reactions towards infertility problems and the consequences of treating this social-clinical problem.
